# Lifting the veil on the keratinocyte contribution to cutaneous nociception

**DOI:** 10.1007/s13238-019-00683-9

**Published:** 2020-01-06

**Authors:** Matthieu Talagas, Nicolas Lebonvallet, François Berthod, Laurent Misery

**Affiliations:** 1 Univ Brest, LIEN, 29200 Brest, France; 2 Laboratoire d’Organogenèse Expérimentale (LOEX), University of Laval, Quebec, Canada; 3 Department of Dermatology, Brest University Hospital, Brest, France; 4 Univ Brest, IBSAM (Institut Brestois de Santé Agro matière), 29200 Brest, France

**Keywords:** keratinocyte, nociception, skin, TRP, pain, inflammation

## Abstract

Cutaneous nociception is essential to prevent individuals from sustaining injuries. According to the conventional point of view, the responses to noxious stimuli are thought to be exclusively initiated by sensory neurons, whose activity would be at most modulated by keratinocytes. However recent studies have demonstrated that epidermal keratinocytes can also act as primary nociceptive transducers as a supplement to sensory neurons. To enlighten our understanding of cutaneous nociception, this review highlights recent and relevant findings on the cellular and molecular elements that underlie the contribution of epidermal keratinocytes as nociceptive modulators and noxious sensors, both under healthy and pathological conditions.

## Introduction

The skin forms a protective and sensory interface between our body and the external environment. Its outermost layer, the epidermis, consists in a stratified squamous epithelium mainly composed of keratinocytes that proliferate from a basal layer over the basement membrane and then differentiate and migrate to the surface in a coordinated way to successively define the spinous and granular layers topped by the stratum corneum.

The dogma underlying somatosensation indicates that keratinocytes account solely for physical and chemical barrier, whereas sensory neurons, via their extremities that pass between keratinocytes and are called intra-epidermal free nerve endings (FNEs), are the exclusive detectors and transducers of noxious thermal, mechanical or chemical stimuli (Woolf and Ma, 2007). This process, referred to as cutaneous nociception, is essential to preserve the individual from injuries by ultimately eliciting a perception of acute pain and the resulting appropriate protective behaviours. Thus, while ambient skin temperature is maintained near 32 °C (Peier et al., 2002a), warm and cold temperatures are respectively perceived as noxious in humans above 42 °C (Caterina et al., 1999; Güler et al., 2002; Peier et al., 2002b) and below 15 °C (Davis and Pope, 2002). Intense pressure, such as pricks, generates painful touch and both environmental noxious and endogenous molecules are detected by chemo-nociceptors. Therefore, peripheral pathological conditions, such as tissue damage or cutaneous inflammation can sensitize the nociceptors by inducing the release of neuroactive substances close to the nerve fibres (McMahon et al., 2008), leading to potentially disabling inflammatory pain with abnormal sensations such as allodynia—innocuous stimuli perceived as painful—or hyperalgesia—normally painful stimuli eliciting a more intense pain than expected.

However, the simplistic opposition between nociceptive sensory neurons and keratinocytes no longer needs to be. Not only can epidermal keratinocytes modulate the transduction in nociceptive sensory neurons, but recent studies have also demonstrated that they can directly initiate nociceptive responses. In the present review, we highlight recent and relevant findings on the cellular and molecular elements that underlie the contribution of epidermal keratinocytes to nociception under both healthy and pathological conditions. We first explore how keratinocytes crosstalk with nociceptive sensory neurons by releasing neuroactive compounds that modulate pain and then partake of an understanding their capacity to sense noxious stimuli as a supplement to the sensory neurons. These findings invite us to reassess the foundation of cutaneous nociception and reveal a new insight into the pathophysiology of pain.

## Classical point of view

**Figure 1:**
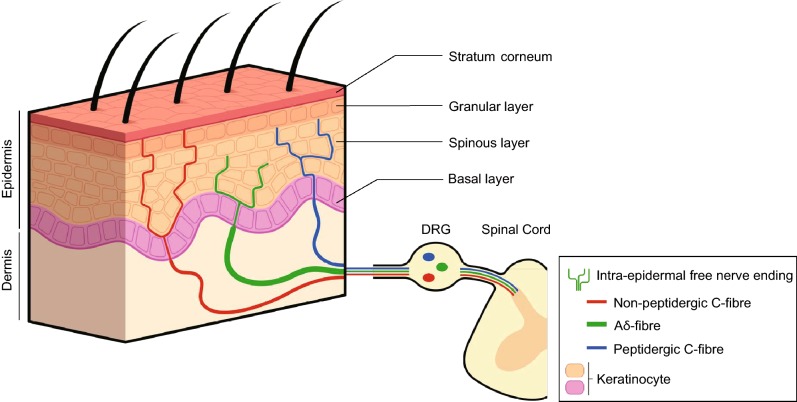
**Intra-epidermal free nerve endings**. The epidermis is innervated by sensory neurons that have cell bodies located in the dorsal root ganglia (DRG) and central projections to the spinal cord. Thinly myelinated Aδ-fibres and unmyelinated C-fibres terminate as intra-epidermal free nerve endings that penetrate to the granular layer of the living epidermis. Aδ-fibres convey the fast and well-localized part of the pain message whereas C-fibres convey the slow and poorly localized part of the pain message. Nociceptive C-fibres are classified into peptidergic C-fibres, which terminate mainly in the spinous layer of the epidermis, and into non-peptidergic C-fibres, which end more superficially in the granular layer

According to the conventional point of view, FNEs are thought to be the sole cutaneous nociceptors (Basbaum et al., 2009). These nerve fibres, which correspond to the dendritic extremities of pseudo-unipolar sensory neurons located in trigeminal and dorsal root ganglia (DRG), ascend and branch in the epidermis, the longest of them ending in the granular layer (Kennedy and Wendelschafer-Crabb, 1993). Nociceptive FNEs are divided into two main categories based on their conduction velocity and their degree of myelination (Abraira and Ginty, 2013). Each of these two categories convey a specific component of the pain message: the fast and well-localized part is transduced by the medium diameter, thinly myelinated Aδ-fibres, whereas the slow and poorly localized part depends on the small diameter, unmyelinated C-fibres (Basbaum et al., 2009). Nociceptive FNEs are mostly polymodal, responding to at least two of the three types—mechanical, thermal, chemical—of noxious stimuli (Baumbauer et al., 2015). Nociceptive C-fibres can be classified into two subpopulations (Snider and McMahon, 1998) that terminate in distinct layers of the epidermis (Zylka et al., 2005). The first one, peptidergic C-fibres, which is regulated by the nerve growth factor (NGF), releases neuropeptides such as substance P (SP) and calcitonin-gene related peptide (CGRP) and terminates mainly in the spinous layer. The second one, non-peptidergic C-fibres, which requires glial-derived neurotrophic factor (GDNF), predominantly expresses Mas-Related G protein-coupled (Mrpg) and purinergic P2X receptors, binds IB4 isolectin (Dong et al., 2001), and ends more superficially in the granular layer (Fig. 1). In addition to these two majors categories, highly myelinated Aβ nociceptors have also been reported (Djouhri and Lawson, 2004).

FNEs transduce noxious stimuli through the activation of specific thermo-, mechano-, or chemo-responsive receptors. Advances in understanding the molecular mechanisms of cutaneous sensory transduction have highlighted the transient receptor potential (TRP) ion channels as the pivotal sensors for nociception, which are involved in both acute pain and inflammatory pain. Since the subject of this review is the contribution of keratinocytes to nociception, we will focus our discussion on these receptors that are also, for the most part, expressed by keratinocytes.

TRP vanilloid 1 (TRPV1), expressed by peptidergic C- and Aδ-fibres (Caterina and Julius, 2001) and activated at temperatures above 42 °C (Caterina et al., 1997), is classically considered the main transducer of noxious heat (Caterina et al., 2000). TRPV2, with a high thermal activation threshold above 52 °C, has emerged as another potential noxious heat transducer (Caterina et al., 1999), but its functionality failed to be proved *in vivo* (Park et al., 2011). In contrast, a recent study has just revealed that TRPV1 mediates acute noxious heat in concert with TRP melastatin 3 (TRPM3) and TRP ankyrin 1 (TRPA1); mice deficient in TRPV1, TRPM3 and TRPA1 showed an almost complete loss of noxious heat responses (Vandewauw et al., 2018).

Environmental cold temperatures are mainly detected by TRPM8 over a wide range from 30 °C to 10 °C (McKemy et al., 2002; Peier et al., 2002a; Bautista et al., 2007; Dhaka et al., 2007), including both innocuous and noxious cold temperatures. In a similar manner to TRPV1 and noxious heat perception, it remains to identify other cold sensors. TRPA1, also activated at around 17 °C and below, and coexpressed with TRPV1 and TRPM3 but not TRPM8, was originally described as a specific noxious cold receptor (Story et al., 2003; Kwan et al., 2006; Karashima et al., 2009; Vandewauw et al., 2018). However, its role in cold acute pain has been controversial (Bautista et al., 2006; Kwan et al., 2009); it has alternatively been described as contributing to cold allodynia and hyperalgesia (Obata et al., 2005; Bautista et al., 2006; del Camino et al., 2010) in response to a large range of environmental pungents irritants such as mustard oil (allyl isothiocyanate), cinnamon oil (cinnamaldehyde), garlic (allicin), and endogenous proalgesic agents produced in the context of tissue damage or cutaneous inflammation such as bradykinin and H_2_O_2_ (Bandell et al., 2004; Jordt et al., 2004; Macpherson et al., 2005; Bautista et al., 2006; Andersson et al., 2008).

Like TRPA1, other TRPs are also polymodal, thus contributing greatly to chemotransduction—both for environmental and endogenous chemicals—and therefore to inflammatory pain. Thus, capsaicin, the main pungent component of chilli peppers (Caterina et al., 1997), and extracellular protons in high concentration (pH < 6) due to tissue injury or inflammation, by binding to TRPV1 and decreasing its temperature threshold activation, make TRPV1 a key contributor to heat allodynia and hyperalgesia (Tominaga et al., 1998; Caterina et al., 2000; Davis et al., 2000). Furthermore, a large range of other endogenous proalgesic agents also produced in response to tissue damage or inflammation, such as bradykinin, prostaglandin, ATP, or NGF (Chuang et al., 2001; Tominaga et al., 2001; Moriyama et al., 2005), can indirectly sensitize TRPV1 by binding to their specific receptors on FNEs and thus elicit hypersensitivity to heat. TRPM3 equally contributes to heat hyperalgesia during inflammation (Vriens et al., 2011), while TRPM8, activated by menthol (Peier et al., 2002a), may participate in the hypersensitivity to cold (Colburn et al., 2007). Changes in pain processing engaged during cutaneous injury or inflammation illustrate that the activity and sensitivity of nociceptive FNEs are influenced by their chemical environment and therefore the surrounding cells, including epidermal keratinocytes.

## Keratinocytes as modulators of nociceptive sensory neurons activity

Keratinocytes are the predominant cells in the epidermis, and the FNEs are in close proximity to them over their entire length (Hilliges et al., 1995), regardless of their subtypes and the layers in which they terminate. As nociceptive C-fibres terminate in distinct epidermal layers according to their nature (Zylka et al., 2005) and keratinocytes progressively differentiate with their migration, these intimate physical contacts provide the opportunity for spatially differentiated paracrine communications between keratinocytes and neurons.

Epidermal keratinocytes can release many neuroactive molecules that can modulate nociception mediated by FNEs, activating or inhibiting sensory neurons. These chemicals include notably neurotrophins such as NGF (Di Marco et al., 1991) and GDNF (Roggenkamp et al., 2012), neuropeptides such as SP (Bae et al., 1999) and CGRP (Hou et al., 2011), ATP (Barr et al., 2013), classical neurotransmitters such as glutamate (Fischer et al., 2009) and acetylcholine (Grando et al., 1993), β-endorphin (Wintzen et al., 1996; Zanello et al., 1999), endothelin-1 (Tsuboi et al., 1995; Khodorova et al., 2002), and cytokines (Shi et al., 2011).

While under healthy conditions, keratinocytes are protective, promoting analgesia (Ji et al., 2016), the balance disruption observed in pathological conditions that promote pain, highlights the pro-nociceptive and anti-nociceptive roles played by keratinocytes within a complex dialogue with sensory neurons. Peptidergic C-fibres also release neuropeptides, particularly SP and CGRP, leading to neurogenic inflammation that contributes, via keratinocyte activation, to amplify their sensitization (Shi et al., 2013).

**Figure 2:**
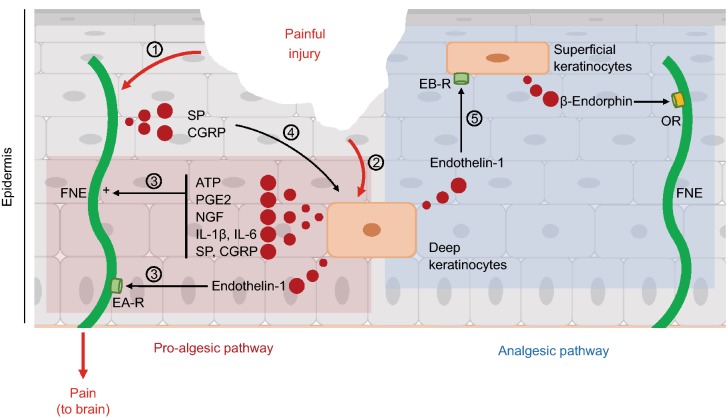
**Epidermal keratinocytes modulate nociceptive sensory neuron activity**. Keratinocytes produce both pro-nociceptive and anti-nociceptive substances, which bind to the intra-epidermal free nerve endings (FNEs) to modulate neuronal activity. (1) Painful cutaneous injury activates nociceptive FNE. (2) Keratinocytes are also exposed to injury. (3) This exposition to injury induces the release of FNE activators by keratinocytes, such as ATP, PGE2, NGF, IL-1β, IL-6, and endothelin-1, which sensitize nociceptive neurons. Endothelin-1 links to endothelin-A receptors (EAR) on sensory neurons (pro-algesic pathway). (4) In return, peptidergic C-fibres release substance P (SP) and CGRP to activate keratinocytes through an amplification loop leading to the neuronal sensitization. (5) Keratinocyte-released endothelin-1 also links to endothelin-B receptors (EBR) on keratinocytes. In response, superficial keratinocytes release β-endorphin, which activates μ- and κ-opioid receptors (OR) in FNEs, and inhibits pain (analgesic pathway)

Because of their superficial localization, epidermal keratinocytes are often the first cells exposed to injuries. In these conditions, damaged keratinocytes excite FNEs due to the release of multiple cytosolic activators of nociceptors such as ATP (Cook and McCleskey, 2002), and protons (Tominaga et al., 1998). Keratinocytes also contribute to neuronal sensitization via NGF, prostaglandin E2, SP, CGRP, interleukins 1β (IL-1β) and 6 (IL-6) or endothelin-1 (Tsuboi et al., 1995; Pei et al., 1998; Li et al., 2009; Radtke et al., 2010; Hou et al., 2011; Shi et al., 2011; Shi et al., 2013). However, keratinocytes play a dual role, and possess an intrinsic feedback mechanism to initiate an analgesic pathway. For example, endothelin-1 triggers pain by linking to endothelin-A receptors on sensory neurons. However, it simultaneously activates endothelin-B receptors on keratinocytes, leading to the secretion of β-endorphin, which activates μ- and κ-opioid receptors in nociceptive FNEs, and ultimately inhibits pain (Khodorova et al., 2003). Moreover, endothelin-1 is mainly released by deeper keratinocytes when β-endorphin is released by the most superficial keratinocytes (Lumpkin and Caterina, 2007). The presence of such spatially distinct antagonist crosstalk points to a complex keratinocyte-FNE communication network modelling nociceptive information from the epidermis level (Fig. 2).

## Keratinocytes as primary nociceptive transducers

The decrease of FNEs density in the human epidermis from the trunk to the extremities without associated loss of sensitivity (Wang et al., 1990; Hilliges et al., 1995; McArthur et al., 1998), is an invitation to cautiously consider sensory neurons as the best-recognized transducers rather than the exclusive cutaneous nociceptors (Talagas et al., 2018a). Because the FNEs are enwrapped by keratinocytes and the longest of them end in the granular layer of the epidermis without reaching the skin surface, epidermal keratinocytes always interpose between the sensory neurons and the environment. Keratinocytes are therefore ideally positioned to transduce environmental stimuli as a supplement to the sensory neurons in a similar fashion as other epithelial cells do, such as hair cells in the auditory system and taste receptor cells in the gustatory system (Finger et al., 2005; LeMasurier and Gillespie, 2005).

Asserting that epidermal keratinocytes act as noxious primary transducers requires demonstrating that they (1) express functional sensory receptors activated by noxious stimuli and (2) induce the release of neuroactive substances that (3) specifically activate nociceptive sensory neurons to ultimately elicit pain. Several arguments explain that the concept of keratinocytes as noxious sensors has only recently emerged. First, for several decades, the dogma underlying cutaneous somatosensation stated that nociceptive sensory neurons were the sole cutaneous nociceptors, thus discouraging the emergence of alternative view. Second, functional receptors harboured by epidermal keratinocytes and classically implicated in noxious perception when expressed by sensory neurons, such as TRP ion channels that include TRPV1 (Denda et al., 2001), TRPV3 (Peier et al., 2002b), TRPV4 (Güler et al., 2002), TRPM8 (Denda et al., 2010a; Bidaux et al., 2015; Bouvier et al., 2018), and TRPA1 (Atoyan et al., 2009), also contribute to the skin homeostasis (Caterina and Pang, 2016). It is the same for neuroactive substances produced by keratinocytes, such as glutamate or ATP that influence the keratinocyte proliferation and differentiation (Greig et al., 2003; Nahm et al., 2004). Third, the complex organisation of the epidermis, with keratinocytes and FNEs intimately associated, makes it impossible to selectively stimulate keratinocytes while ignoring sensory neurons. However, the advent of tools such as keratinocyte-sensory neuron cocultures modelling neuro-epithelial interactions and opto- and chemogenetic transgenic mouse models allowed us to overcome this pitfall and assert that keratinocytes can initiate nociceptive transduction.

### Acute pain

With regards to Merkel cells and innocuous mechanotransduction (Maksimovic et al., 2014), a proof of concept in acute pain has been provided by an optogenetic mouse model that targets channelrhodopsin (ChR2), a blue light-gated cation channel. Light stimulation of ChR2, when exclusively expressed by epidermal keratinocytes, is sufficient to induce action potentials in specific subsets of sensory neurons, i.e., Aδ-, C-, and Aβ-nociceptors, and so elicit nocifensive behaviours (Baumbauer et al., 2015). Therefore, epidermal keratinocytes and nociceptive sensory neurons may act as a two-receptor-site model, each conveying specific aspects of the nociceptive information, similar to the behaviour of Merkel cells and Aβ- nerve fibres in Merkel complexes (Ikeda et al., 2014). Indeed, cutaneous blue-light exposure of mice expressing ChR2 only in sensory neurons does generate protective behaviours and action potential firings in nociceptive fibres, but their response profiles differ from those induced by inartificial noxious stimuli. Thus, for example, C-nociceptors exhibit a tonic response to noxious mechanical stimulation, whereas blue light evokes a more phasic response, suggesting that natural firing pattern requires the collaboration of nociceptive neurons and surrounding keratinocytes (Baumbauer et al., 2015).

**Figure 3:**
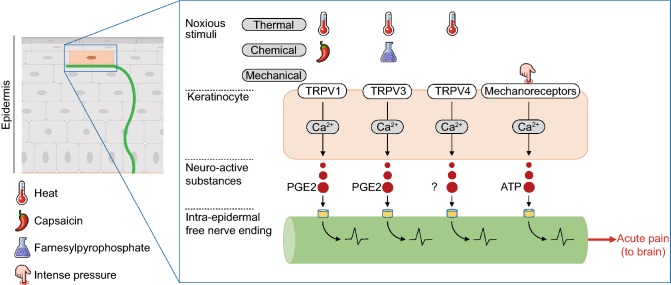
**Epidermal keratinocytes initiate acute pain**. Epidermal keratinocytes express functional sensory receptors, such as TRPV1, TRPV3, TRPV4, and mechanoreceptors (not yet identified). Their activation by noxious stimuli causes a calcium-dependant release of neuroactive substances that specifically activate nociceptive sensory neurons to ultimately elicit acute pain. The substance released in response to TRPV4 activation has not been yet identified. TRPV3 and TRPV4 are probably not major noxious thermosensors (Huang et al., 2011)

These results, together with two other recent findings based on conventional noxious stimuli (Pang et al., 2015; Moehring et al., 2018a) call for reconsideration in the field of cutaneous nociception and shed new light on previously conducted studies. As this paradigm shift emerged initially after the identification of TRP ion channels in keratinocytes, we describe at first scientific advances related to each of these heat- and then cold-sensitive sensory receptors, before continuing with the keratinocyte contribution to mechanical nociception (Fig. 3).

The expression of functional TRPV1 in human keratinocytes, where capsaicin and protons that induce an increase in the intracellular calcium concentration lead to the release of interleukin-8 and prostaglandin E2 (PGE2), provides a first argument for the contribution of this keratinocyte-expressed channel to noxious perception (Denda et al., 2001; Inoue et al., 2002; Southall et al., 2003). This information is in line with recent findings indicating that the stimulation of keratinocyte-expressed TRPV1 is sufficient to activate nociceptive sensory neurons and trigger acute pain (Pang et al., 2015). Thus, cutaneous applications of capsaicin in *Trpv1* knockout mice genetically conFig.d to exclusively express TRPV1 in epidermal keratinocytes induced nocifensive behaviours and a strong expression of the neuronal activation marker c-fos in laminae I and II of the spinal cord dorsal horn, both receiving nociceptive information from the skin. As capsaicin could only activate keratinocyte-expressed TRPV1, keratinocyte acted as primary nociceptive transducers that selectively stimulated downstream FNEs. It should also be noted that both peptidergic and non-peptidergic nociceptive neurons were involved, whereas in wild-type mice, c-fos expression, secondary to the application of capsaicin, predominated in the peptidergic population, in accordance with TRPV1 neuronal distribution (Caterina and Julius, 2001). This suggests that epidermal keratinocytes could shape the nociceptive message by selectively activating non-peptidergic FNEs, which end more superficially in the epidermis, in addition to peptidergic FNEs. Such epidermal layer-specific dialogue would support the concept of a two-receptor-site model mentioned above.

The persistence of significant responses to acute noxious heat in *Trpv1* knockout mice (Caterina et al., 2000; Davis et al., 2000) as well as in skin-nerve *ex vivo* preparations derived from *Trpv1* knockout mice (Woodbury et al., 2004; Zimmermann et al., 2005), but not found in sensory neurons lacking TRPV1 *in vitro* (Pogorzala et al., 2013), suggests that additional sensors, preferably expressed by other cell types, might participate in noxious heat transduction. Thus, TRPV3 and TRPV4, which are activated at approximately 33 °C and 27 °C, respectively (Güler et al., 2002; Peier et al., 2002b), described to sense both innocuous and noxious heat (Güler et al., 2002; Peier et al., 2002b; Smith et al., 2002) and predominantly expressed by keratinocytes compared to sensory neurons (Peier et al., 2002b; Xu et al., 2002; Lumpkin and Caterina, 2007), first appeared as the most promising candidates for TRPV1-independent noxious heat transduction. This reflexion supported an argument in favour of keratinocyte contribution to nociceptive transduction. Both TRPV3 and TRPV4 mediate noxious heat-evoked currents in mouse keratinocytes (Chung et al., 2004). Moreover, noxious heat (43 °C) activation of TRPV3 causes a calcium- and cyclooxygenase-1 (COX1)-dependent release of PGE2 (Huang et al., 2008), as also observed for TRPV1 (Southall et al., 2003). Its chemical activation with farnesyl pyrophosphate, a specific endogenous activator involved in the cholesterol synthesis pathway, results in neuronal activation (Bang et al., 2010). Behavioural studies of mice deficient in TRPV3 or TRPV4 have reinforced this hypothesis. *Trpv3* knockout mice showed delayed nocifensive responses at 50 °C and above (Moqrich et al., 2005) comparable to those of TRPV1-deficient mice. Conversely, the selective keratinocyte overexpression of TRPV3 in transgenic mice was associated with an accentuation of acute pain, but only in the presence of TRPV1 inhibitor (Huang et al., 2008). A deficit was also present in *Trpv4* knockout mice, but it was slight and restricted to 45 °C and 46 °C (Lee et al., 2005). Although encouraging, these results also indicate that TRPV3 and TRPV4 are probably not major noxious thermosensors. By revealing that mice lacking both TRPV3 and TRPV4 showed slightly delayed withdrawals responses only for a type of pain-heat assay, a most recent study confirmed the modest but also genetic background-dependent contribution of TRPV3 and TRPV4 to acute noxious heat perception (Huang et al., 2011).

Consistent with these observations, and as discussed above, TRPA1 and TRPM3 have recently been identified, in addition to TRPV1, in *Trpv1*^−/−^*Trpm3*^−/−^*Trpa1*^−/−^ triple knockout mice, as additional members of a major trio involved in acute noxious heat sensing (Vandewauw et al., 2018). However, data concerning TRPA1 and TRPM3 in keratinocytes are lacking. Human epidermal keratinocytes express TRPA1 (Atoyan et al., 2009), but its contribution to noxious heat transduction has not been investigated yet, in contrast to its role in noxious cold. Moreover, to our knowledge, TRPM3 expression in keratinocytes has not been explored (Oberwinkler and Philipp, 2014).

Additionally, the findings from global knockout mice, whether for TRPV3 and TRPV4 on the one hand or for TRPA1 and TRPM3 on the other hand, do not allow us to specifically appreciate the contribution of epidermal keratinocytes. Keratinocyte and sensory neuron selective knockouts might also be helpful to obtain more conclusive information.

Although TRPM8 is expressed by human keratinocytes (Denda et al., 2010a; Bidaux et al., 2015; Bouvier et al., 2018), little is known about its functions. Keratinocyte-expressed TRPM8 does act as a cold sensor, at least to control the balance between keratinocyte proliferation and differentiation (Bidaux et al., 2015). Similarly, keratinocyte-expressed TRPA1 (Atoyan et al., 2009) induces elevation of intracellular calcium and accelerates epidermal recovery in a cold-dependent manner (Denda et al., 2010b; Tsutsumi et al., 2010). However, their contributions to cold nociception, via neuronal activation and beyond nocifensive behaviours, have not been yet investigated.

Recent data also indicate that epidermal keratinocytes can transduce noxious mechanical stimuli. Thus, in an optogenetic mouse model inspired by Baumbauer et al. (2015), light-stimulation of archaerhodopsin-3 (Arch)—a cation channel inducing cell membrane hyperpolarization—reduces action potentials in C-fibres and inhibits nociceptive responses to noxious mechanical stimuli when exclusively expressed by epidermal keratinocytes (Moehring et al., 2018a). Moreover, this study identifies keratinocyte-released ATP as a key mediator, activating P2X4 receptors on sensory neurons to elicit nocifensive behaviours. Admittedly, mechanical induced release of ATP from keratinocytes and corollary neuronal activation have been previously reported *in vitro*, but the innocuous or noxious nature of the stimulation was not specified (Koizumi et al., 2004; Tsutsumi et al., 2009). Contrary to thermo- and chemotransductors, keratinocyte mechanosensors remain poorly known to date (Moehring et al., 2018b). Nevertheless, it appears that mice deficient for TRPV4 exhibit impaired acute mechanical nociception (Suzuki et al., 2003).

### Inflammatory pain

**Figure 4:**
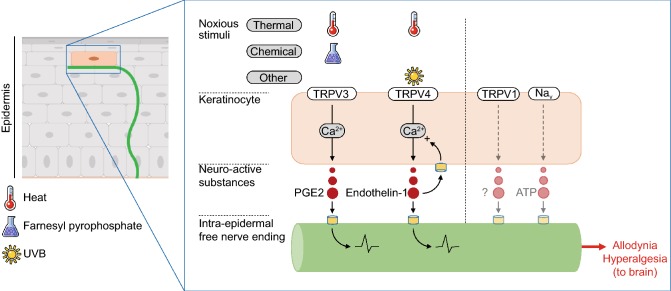
**Epidermal keratinocytes initiate inflammatory pain**. Epidermal keratinocytes express functional TRPV3 and TRPV4 activated by noxious heat, inducing the release of neuroactive substances that specifically activate nociceptive sensory neurons to ultimately elicit allodynia and hyperalgesia. TRPV3 can also be activated by farnesyl pyrophosphate. Keratinocyte TRPV1 and Na_v_ overexpression has also been reported in patients suffering from chronic pain, but the functionality has not been evaluated

Epidermal keratinocytes can also act as primary transducers in inflammatory pain, which appears to be attributable to TRPV4 (Fig. 4). Thus, thermal and mechanical allodynia induced by acute UVB exposure is reduced in a sunburn mouse model deficient in TRPV4 exclusively in the epidermal keratinocytes (Moore et al., 2013). Moreover, there is no major hypersensitivity attenuation in *Trpv4* global knockout mice, despite the additional neuronal TRPV4 deficit, indicating the key role of keratinocytes. The activation of keratinocyte-expressed TRPV4 by UVB is necessary and sufficient to elicit pain via the release of endothelin-1 and induce epidermal damage. In return, endothelin-1 amplifies TRPV4 calcium influx in keratinocytes, and consequently results in allodynia, due to an autocrine and paracrine amplification loop. In accordance with these observations, TRPV4 and endothelin-1 expression is increased in the human epidermis of sunburned patients (Moore et al., 2013). Furthermore, this elegant study sheds new light on the reduced thermal and mechanical hyperalgesia previously reported in Trpv4 global knockout mice and mice treated with TRPV4 antisense oligonucleotides (Alessandri-Haber et al., 2004; Todaka et al., 2004). In addition, no defect in heat hyperalgesia was observed in a mouse model lacking both TRPV3 and TRPV4 (Huang et al., 2011), suggesting a requirement for another factor such as endothelin 1.

The TRPV3 contribution to hypersensitivity remains less clear, as not reduction in heat hyperalgesia was reported in mice deficient for TRPV3 or both TRPV3 and TRPV4 (Moqrich et al., 2005; Huang et al., 2011). However, support for the role of TRV3, most likely modest, has come from behavioural assays of mice overexpressing TRPV3 exclusively in keratinocytes (Huang et al., 2008). Under inflammatory conditions, these mice exhibit delayed responses in noxious heat behavioural assays compared to wild-type mice, but only after the administration of ibuprofen, a cyclooxygenase inhibitor, which disrupts the PGE2 synthesis. Furthermore, dermal injections of farnesyl pyrophosphate, an endogenous agonist of TRPV3, elicit allodynia and hyperalgesia in inflamed mice. However, specific inactivation of TRPV3 in keratinocytes might provide more definitive arguments, in a similar manner to keratinocyte-expressed TRPV4.

### Voltage-gated sodium channels

The stimulation of TRP ion channels induces calcium influx but also membrane depolarization both in sensory neurons and keratinocytes (Caterina and Pang, 2016). In sensory neurons, the depolarization activates voltage-gated sodium channels (Na_v_), which are essential to triggering action potential firing, but also involved in the pathogenesis of neuropathic pain (Waxman et al., 2000). Although non-excitable cells, epidermal keratinocytes also express several Na_v_ isoforms, such as Na_v_ 1.1, Na_v_ 1.2, Na_v_ 1.5, Na_v_ 1.6, Na_v_ 1.7 and Na_v_ 1.8 (Zhao et al., 2008). Because they contribute to the keratinocyte release of ATP in a depolarization-dependent manner, thus potentially activating sensory neurons downstream, and because they are overexpressed by the keratinocytes of patients suffering from complex regional syndrome type 1 (CRPS) and post-herpetic neuralgia (PHN), epidermal keratinocytes may also contribute to chronic pain via Na_v_, in a similar manner to sensory neurons do (Zhao et al., 2008).

Keratinocyte TRPV1 expression was also found to be increased in herpes zoster patients and in a rodent model of immobilization-induced pain, suggesting that excessive nociceptive transduction may occur in keratinocytes (Sekino et al., 2014; Han et al., 2016). A definitive evaluation of the contribution of keratinocyte-expressed Na_v_ and TRPV1 to chronic pain calls for additional *in vivo* functional approaches.

## Conclusion and perspectives

The identification of keratinocytes as primary noxious transducers is a paradigm shift in the field of cutaneous sensory transduction. This renewed conception invites the consideration of the whole epidermis as a sensory epithelium (Boulais and Misery, 2008). Our understanding is just emerging, providing a fascinating insight into the respective contribution of keratinocytes and noxious sensory neurons in the initiation of nociceptive responses. The well-admitted modulation conducted by keratinocytes on sensory neuron activity could result, at least in part, from their capacity to transduce noxious information. Furthermore, this new knowledge already points to the incredible complexity of cutaneous cellular interactions necessary to shape relevant noxious information to the nervous system. The next challenge is to decipher, with molecular and functional approaches, the language shared by the two protagonists for distinctly encoding noxious stimuli. Neuronal and keratinocyte dysfunctions could contribute to pathological pain, opening new potential therapeutic target for pain.

These matching discoveries both in normal and pathological conditions imply the existence of close afferent communications from keratinocytes to FNEs in order to ensure a specific subset neuronal activation and ultimately an adequate painful perception. However, the histological, functional and molecular characteristics of keratinocyte-FNE communications remain poorly understood. Another future key challenge is to discover the mechanism(s) that underlie(s) the sensory dialogue between epidermal keratinocytes and sensory neurons. In the Merkel complex, synaptic contacts between Merkel cells and Aβ-fibres ensure the speed and specificity required for communication (Haeberle et al., 2004; Maksimovic et al., 2013). Because keratinocytes descend from epidermal stem cells that also give rise to Merkel cells (Morrison et al., 2009; Van Keymeulen et al., 2009) it is tempting to hypothesize that they could also communicate with sensory neurons via synaptic structures (Talagas et al., 2018b). The close contacts between keratinocytes and FNEs, propitious to rapid paracrine communication (Cauna, 1973; Hilliges et al., 1995), and physical contacts reported as essential to convey sensory information from keratinocytes to sensory neurons in a coculture model (Sondersorg et al., 2014) may lend weight to this idea.
